# Enhanced Performance of Perovskite Single-Crystal Photodiodes by Epitaxial Hole Blocking Layer

**DOI:** 10.3389/fchem.2020.00791

**Published:** 2020-09-10

**Authors:** Yuzhu Pan, Xin Wang, Yubing Xu, Yuwei Li, Elias Emeka Elemike, Ahmed Shuja, Qing Li, Xiaobing Zhang, Jing Chen, Zhiwei Zhao, Wei Lei

**Affiliations:** ^1^School of Electronic Science and Engineering, Joint International Research Laboratory of Information Display and Visualization, Southeast University, Nanjing, China; ^2^Chemistry Department of North West University, Mafikeng, South Africa; ^3^Centre for Advanced Electronics and Photovoltaic Engineering, International Islamic University, Islamabad, Pakistan

**Keywords:** perovskite single crystals, heterojunction, PIN diodes, epitaxial growth, photoelectric detection

## Abstract

Introducing hole/electron transporting and blocking layers is considered to enhance the performance of electronic devices based on organic–inorganic hybrid halide perovskite single crystals (PSCs). In many photodiodes, the hole/electron transporting or blocking materials are spin-coated or thermal-evaporated on PSC to fabricate heterojunctions. However, the heterojunction interfaces due to lattice mismatch between hole/electron, transporting or blocking materials and perovskites easily form traps and cracks, which cause noise and leakage current. Besides, these low-mobility transporting layers increase the difficulty of transporting carriers generated by photons to the electrode; hence, they also increase the response time for photo detection. In the present study, MAPbCl_3_-MAPbBr_2.5_Cl_0.5_ heterojunction interfaces were realized by liquid-phase epitaxy, in which MAPbBr_2.5_Cl_0.5_ PSC acts as an active layer and MAPbCl_3_ PSC acts as a hole blocking layer (HBL). Our PIN photodiodes with epitaxial MAPbCl_3_ PSC as HBL show better performance in dark current, light responsivity, stability, and response time than the photodiodes with spin-coated organic PCBM as HBL. These results suggest that the heterojunction interface formed between two bulk PSCs with different halide compositions by epitaxy growth is very useful for effectively blocking the injected charges under high external electric field, which could improve the collection of photo-generated carriers and hereby enhance the detection performance of the photodiode. Furthermore, the PIN photodiodes made of PSC with epitaxial HBL show the sensitivities of 7.08 mC Gy_air_^−1^ cm^−2^, 4.04 mC Gy_air_^−1^ cm^−2^, and 2.38 mC Gy_air_^−1^ cm^−2^ for 40-keV, 60-keV, and 80-keV X-ray, respectively.

**Graphical Abstract:**
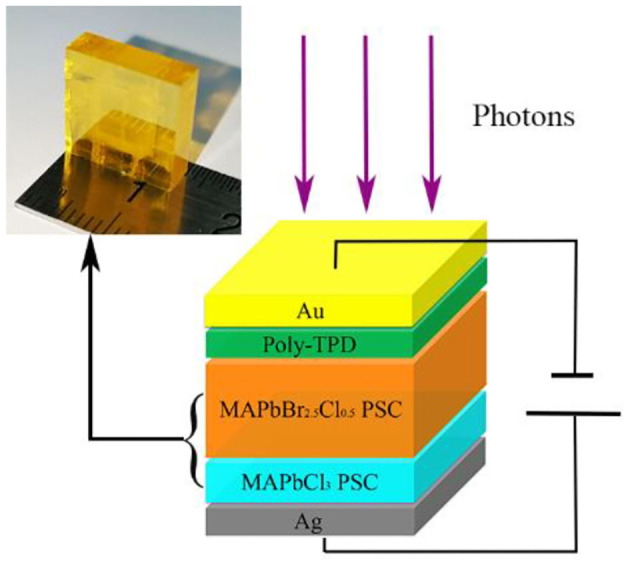
Stratiform MAPbBr_2.5_Cl_0.5_/MAPbCl_3_ heterojunction perovskite single crystal is fabricated by epitaxy growth and then poly-TPD is spin-coated on the face of MAPbBr_2.5_Cl_0.5_ to form an electron blocking layer. Interestingly, the PIN perovskite photodiode-based epitaxial hole blocking layers reveal higher photoelectric properties and satisfactory detectability for 40- to 80-keV X-ray.

## Introduction

Perovskite is a promising candidate among new-generation opto-electronic material for applications in solar cells and light-emitting diodes owing to its outstanding optical and electrical characteristics (Kojima et al., 2009; Tan et al., 2014; Ju et al., 2018). In particular, organic–inorganic hybrid halide lead single crystal perovskites (MAPbX_3_, where MA = CH_3_NH_3_ and X = Cl, Br, or I) with high atomic numbers have been studied. Excellent carrier mobility, low trap density, long carrier diffusion length, and high absorption coefficient indicate that they could be applied not only in regular ultraviolet, visible, and near-infrared light, but also in X-ray and gamma-ray detection and imaging (Wang et al., 2015; Yakunin et al., 2015; Wei et al., 2016; Gill et al., 2018). For perovskite photodiodes, the vertical (sandwich-like) topology structures generated by introducing hole/electron transporting or blocking layers are considered to enhance the performance (Wang and Kim, 2017; Zeng et al., 2017). In many devices based on perovskite crystal, n-type materials including fullerene-derivative phenyl-C61-butyric acid methyl ester (PCBM), ZnO, and TiO_2_ act both as electron transport layers (ETL) and hole blocking layers (HBL). The p-type polymers including poly[N,N′-bis(4-butylphenyl)-N,N′-bis(phenyl)-benzidine] (poly-TPD), poly(ethylenedioxythiophene):polystyrene sulfonate (PEDOT:PSS), poly(9-vinlycarbazole) (PVK), and poly[bis(4-phenyl)(2,4,6-trimethylphenyl)amine] (PTAA) are used both as hole transport layers (HTLs) and electron blocking layers (EBLs) (Dai et al., 2014; Jara et al., 2014; Liu and Kelly, 2014; Malinkiewicz et al., 2014).

The intimate contact between the active layer and hole/electron blocking layers is essential for blocking injected charges and effectively collecting carriers to the electrodes (Chen et al., 2017; Cheng et al., 2019). However, these additional layered transporting and blocking layers deposited by spin-coating or thermal evaporation have a low mobility lifetime and high trap density because these layers are amorphous and have lattice mismatch on PSC interfaces (Lin et al., 2015; Zhang et al., 2017). In addition, surface traps or cracks are easily formed when they deposit large-area and 10-nm-thick functional layers that would cause large noise and leaking current (Fang and Huang, 2015; Wang et al., 2019). MAPbBr_2.5_Cl_0.5_ PSC has been proven to possess large resistivity, high mobility, and low degree of lattice mismatch as compared to MAPbCl_3_ PSC (Sutherland and Sargent, 2016; Jiang et al., 2019; Ou et al., 2019). Therefore, it is feasible to fabricate lattice-matched heterojunctions with the energy band gradient between MAPbBr_2.5_Cl_0.5_ PSC and MAPbCl_3_ PSC by epitaxy growth. This would significantly decrease the leaking current and accelerate carrier transport, leading to a high-performance PSC photodetector (Li et al., 2019b).

This article demonstrates a facile process for fabricating stratiform MAPbBr_2.5_Cl_0.5_/MAPbCl_3_ heterojunction PSCs by liquid-phase epitaxial growth. MAPbBr_2.5_Cl_0.5_ PSC mainly acts as an active layer to absorb photons and n-type MAPbCl_3_ PSC as HBL to decrease the positive charges injected from the anode. Subsequently, p-type organic molecules poly-TPD are deposited on the opposite faces of doped MAPbBr_2.5_Cl_0.5_ PSC to form HTL to block the negative charges injected from the cathode. Finally, the gold and silver films are deposited on the faces of poly-TPD and MAPbCl_3_ PSC as anode and cathode, respectively. The device with electron transport material PCBM and C60 as HBL was fabricated by spin coating to compare with our device. Our PIN photodiode supplanted organic micro molecule with MAPbCl_3_ PSC acting as HBL on MAPbBr_2.5_Cl_0.5_ PSC shows a lower dark current density, greater responsivity, and faster response time. It demonstrates the superiority of taking lattice matched heterojunction by epitaxy growth in the fabricating of perovskite diode. Furthermore, the PIN photodiode with epitaxial MAPbCl_3_ PSC as HBL also shows excellent performance on low-energy X-ray detection due to it being a few millimeters in thickness.

## Materials and Methods

### Materials

Methylamine ethanol solution (CH_3_NH_2_, 33 wt.%), hydrobromic acid (HBr, 48 wt.%), hydrochloric acid (HCl, 37 wt.%), poly-TPD, PCBM, and C60 (99.99%) were obtained from Aladdin. N,N-Dimethylformamide (DMF) and dimethyl sulfoxide (DMSO) were obtained from Alfa Aesar. Lead chloride (PbCl_2_, 99.9%) and lead bromide (PbBr_2_, 99.9%) were purchased from Sigma-Aldrich. All commercial products were used as received.

### PSC Growth

To synthesize MACl and MABr, 1 mol L^−1^ HCl and 1 mol L^−1^ HBr were poured into 1 mol L^−1^ methylamine ethanol solution. Powder-like MACl and MABr were obtained after drying in vacuum at 150°C. To prepare the precursor solutions of MAPbCl_3_, 4.05 g (1 mol L^−1^) of MACl, 16.72 g (1 mol L^−1^) of PbCl_2_, and 45 ml of DMSO were dissolved in 60 ml of DMF. To prepare the precursor solutions of MAPbBr_2.5_Cl_0.5_, 6.72 g (1 mol L^−1^) MABr, 16.52 g (0.75 mol L^−1^) of PbBr_2_, and 4.17 g (0.25 mol L^−1^) of PbCl_2_ were dissolved in 60 ml of DMF. The solutions were filtered by a polytetrafluoroethylene (PTFE) filter with a 30-μm pore size. The filtrate was then transferred to a culture dish and placed on a programmable heating station (IKA-RET control-visc). For MAPbCl_3_ PSC, the temperature was first set at 45°C and raised by 0.2°C h^−1^ until it reached 60°C. For epitaxial and pristine MAPbBr_2.5_Cl_0.5_ PSC, the temperature was first set at 50°C and raised by 0.2°C h^−1^ until it reached 65°C.

### Device Fabrication

To fabricate epitaxial EBL photodiodes, 100-nm poly-TPD and 50-nm Au electrodes were deposited on the face of epitaxial MAPbBr_2.5_Cl_0.5_ PSCs, and 50-nm Ag electrodes were deposited on the face of MAPbCl_3_ PSCs by thermal evaporation in vacuum. For the spin-coated EBL device, 100-nm poly-TPD and a 50-nm Au electrode were deposited on one face of pristine MAPbBr_2.5_Cl_0.5_ PSC by thermal evaporation in vacuum and 10-nm C60 and 50-nm PCBM were deposited on the opposite face by spin coating at 1,000 r min^−1^ in 15 s. Subsequently, Ag electrodes were deposited on it by thermal evaporation in vacuum. To optimize the epitaxial PIN photodiode surface, a diamond wire with a diameter of 0.08 mm was used to remove the extra surrounding at a sawing speed of 3,000 r min^−1^.

### Measurements

XRD patterns were obtained by X'TRA (Switzerland). SEM images and EDX were obtained by Quanta 200 FEI (USA). PL (photoluminescence) patterns were obtained by Edinburgh instruments FS5 (UK). Dark current density–voltage (*J*–*V*) characteristics were measured by a Keithley 4,200 semiconductor analyzer in darkness. The response time and transmit time were measured using an Agilent oscilloscope with a Keithley 2,400 as the voltage source and a 365-nm pulsed Nd:YAG laser with 6-ns pulse width at 20 Hz as the illumination source. Responsivity spectra were measured using Zolix tunable 500-W xenon arc lamp light as the illumination source and a Keithley 4,200 semiconductor analyzer. The photocurrent in X-ray detection performance was measured by a Keithley 6,487 Pico ammeter, and the X-ray dose rate was obtained by a commercial dosimeter (FJ-347A, China). The X-ray source was provided by Nanjing Perlove Medical Equipment Company.

## Results and Discussion

**Figure 1:**
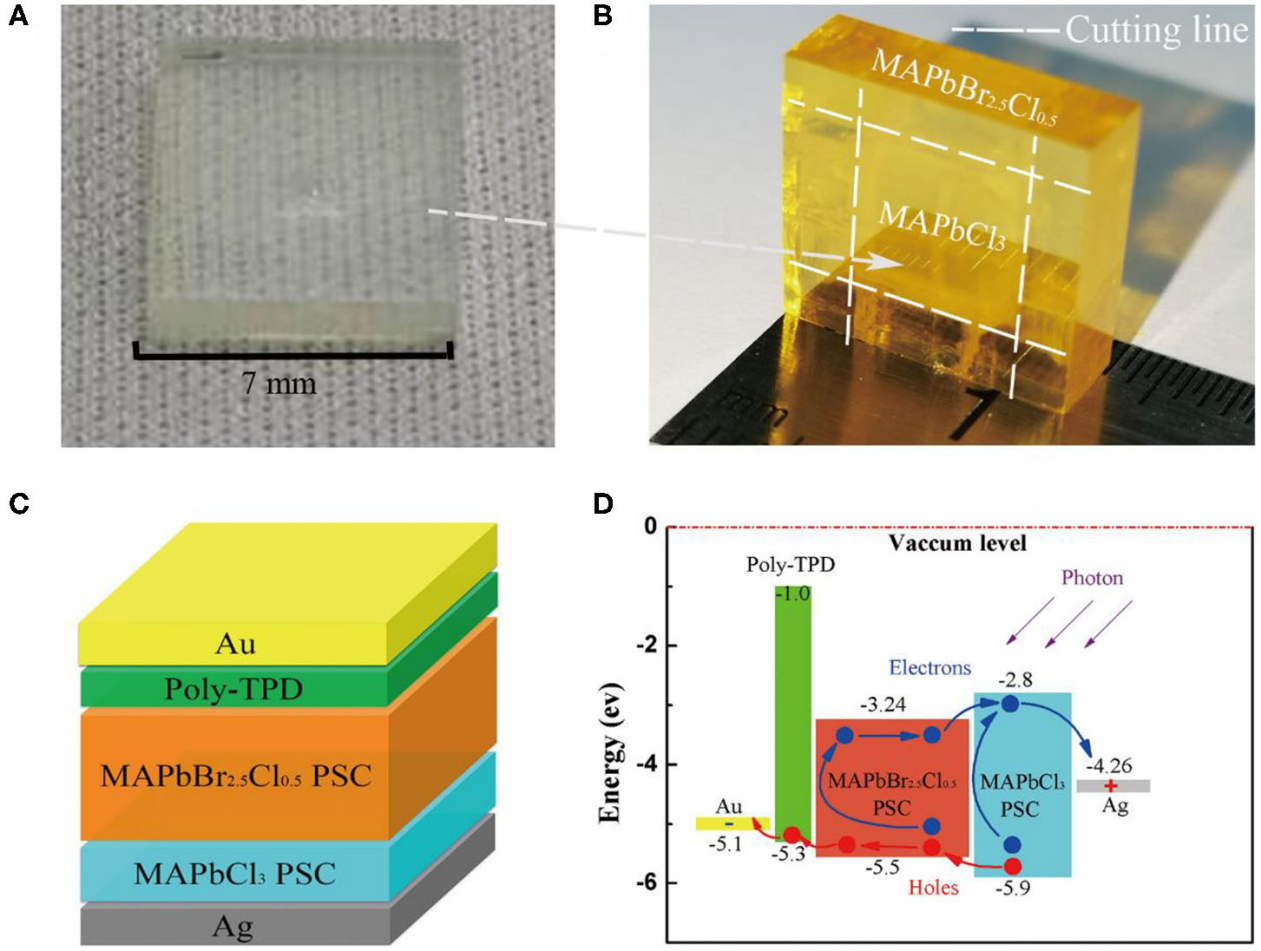
**(A)** Optical image of the colorless and transparent MAPbCl_3_ PSC grown by solution processes. **(B)** Optical image of the yellow MAPbBr_2.5_Cl_0.5_ enfolding the transparent MAPbCl_3_ PSC after epitaxy growth. **(C)** Schematic representation of the full photodiode structure after cutting the surrounding. **(D)** Energy level diagram of the PIN photodiode with epitaxial HBL.

Pristine MAPbCl_3_ PSCs considered as n-type semiconductors were grown by various temperature crystallization methods. The precursor solution was heated from 45 to 60°C for 80 h to grow MAPbCl_3_ PSCs (Wang et al., 2017). First, one unit in bulk of MAPbCl_3_ PSCs was synthesized by low-cost solution processes, as shown in Figure 1A. Then, it was placed into the precursor solution of MAPbBr_2.5_Cl_0.5_ to induce liquid-phase epitaxial growth, in which the solution was heated from 50 to 65°C for 100 h. The MAPbBr_2.5_Cl_0.5_ PSC slowly grew on the top and side of MAPbCl_3_ in the precursor solution. Finally, the heterojunction PSC could be extracted from the solution with MAPbBr_2.5_Cl_0.5_ enfolding MAPbCl_3_ PSC as shown in Figure 1B. In order to fabricate the PIN photodiode, p-type poly-TPD thin film was deposited on the surface of the MAPbBr_2.5_Cl_0.5_ PSC. Subsequently, Au and Ag thin film was deposited on the poly-TPD layer and the surface of MAPbCl_3_ PSC sequentially by thermal evaporation in vacuum.

Figure 1C shows the structure of the fabricated PIN photodiodes after cutting the surrounding, which is Au/poly-TPD/MAPbBr_2.5_Cl_0.5_ PSC/MAPbCl_3_ PSC/Ag. Poly-TPD serves as both HTL and EBL. MAPbCl_3_ PSC serves as both ETL and HBL (Sutherland and Sargent, 2016). Au and Ag function as the anode and cathode, respectively. The schematic energy level diagram of the PIN photodiode is shown in Figure 1D, in which the photo-generated electrons and holes in the active layer are effectively separated and transported to their corresponding electrodes under external electric field. Meanwhile, poly-TPD and MAPbCl_3_ PSC can effectively block the injected electrons and holes from the applied voltage source, respectively, benefiting the reduction of the dark current. The energy barriers of 4.1 eV and 1.64 eV between Au and poly-TPD and between MAPbCl_3_ PSC and Ag significantly reduce the injection of electrons and holes from the anode and cathode, respectively. The energy offset between poly-TPD and the conduction band minimum (CBM) of MAPbBr_2.5_Cl_0.5_ PSC is sufficiently large to block the transfer of electrons from MAPbBr_3_ PSC to the cathode.

**Figure 2:**
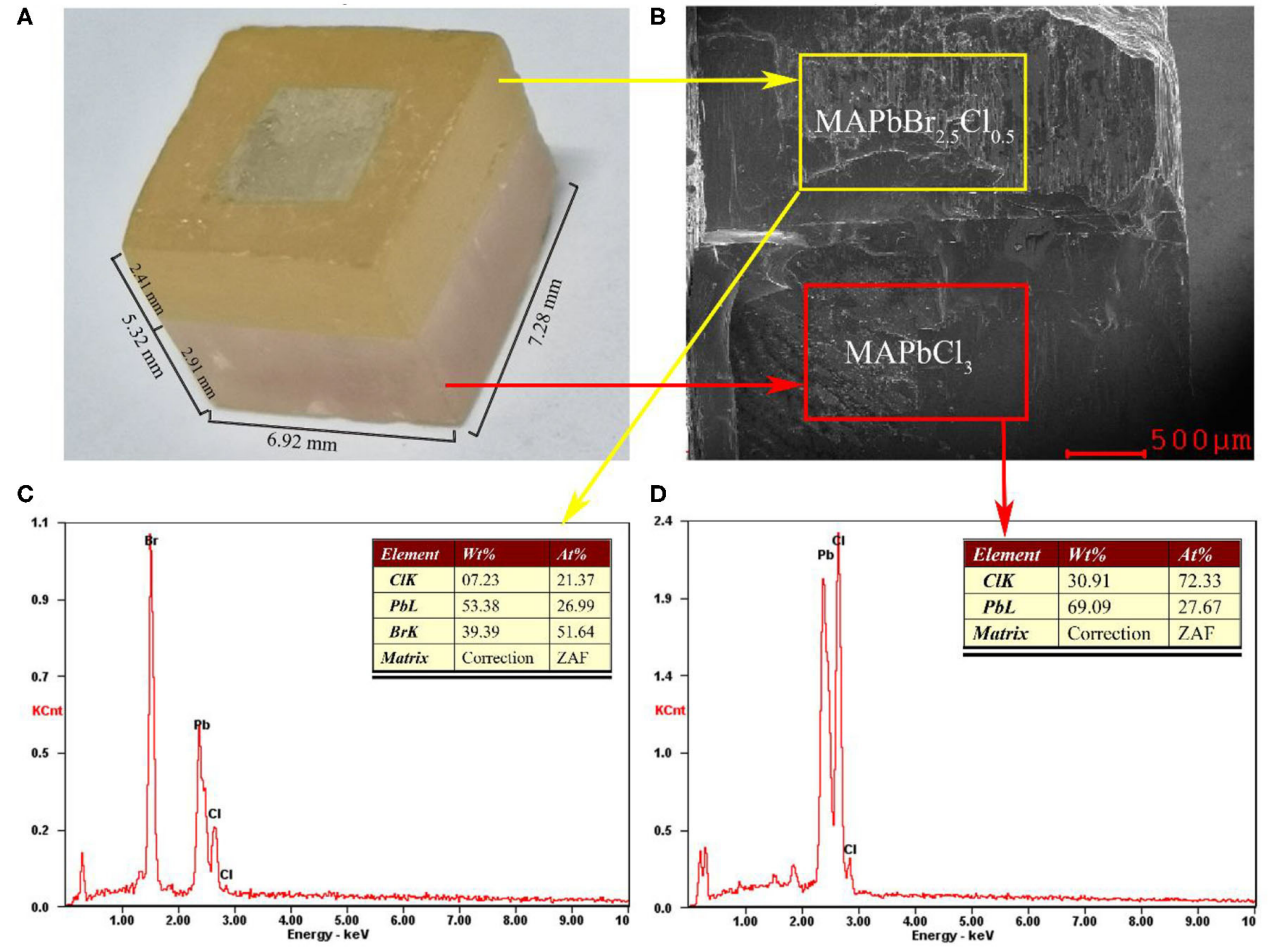
**(A)** Optical image of the epitaxial PIN device. **(B)** Cross-sectional SEM images of the double-layer perovskite bulk. EDX spectra of **(C)** epitaxial MAPbBr_2.5_Cl_0.5_ perovskite layer and **(D)** MAPbCl_3_ perovskite layer.

To optimize the crystal surface, we utilized a diamond wire to saw off the extra surrounding of the MAPbBr_2.5_Cl_0.5_/MAPbCl_3_ heterojunction PSC bulk along the cutting lines shown in Figure 1B. The optical photograph of our PIN photodiode after machining the surrounding is shown in Figure 2A. The dimensions of our PIN photodiode are 7.28, 6.92, and 5.32 mm in length, width, and thickness, respectively. The effective electrode contact area is approximately (4.17 × 5.26 mm) 21.93 mm^2^. Moreover, the thicknesses of the MAPbBr_2.5_Cl_0.5_ intrinsic layer and MAPbCl_3_ HBL are approximately 2.41 and 2.91 mm, respectively.

In this study, we have utilized energy dispersive X-ray (EDX) spectroscopy to investigate the lead and halide element distribution of MAPbCl_3_ PSC on epitaxial MAPbBr_2.5_Cl_0.5_. Figure 2B shows the cross-sectional scanning electron micrograph (SEM) image of the epitaxial perovskite bulk. Two parts of different halide content in the bulk are selected to analyze the EDX spectra. The EDX spectra of the epitaxial MAPbBr_2.5_Cl_0.5_ (areas outlined in yellow) and MAPbCl_3_ (areas outlined in red) are shown in Figures 2C,D, respectively. The ratios of the Pb, Br, and Cl elements in the two kinds of perovskite layers reveal a slight difference in the ratios of the precursor solution. This can be attributed to the considerable difference in the solubility of MABr/Cl and PbBr_2_/Cl_2_ in DMF (Wei et al., 2016).

**Figure 3:**
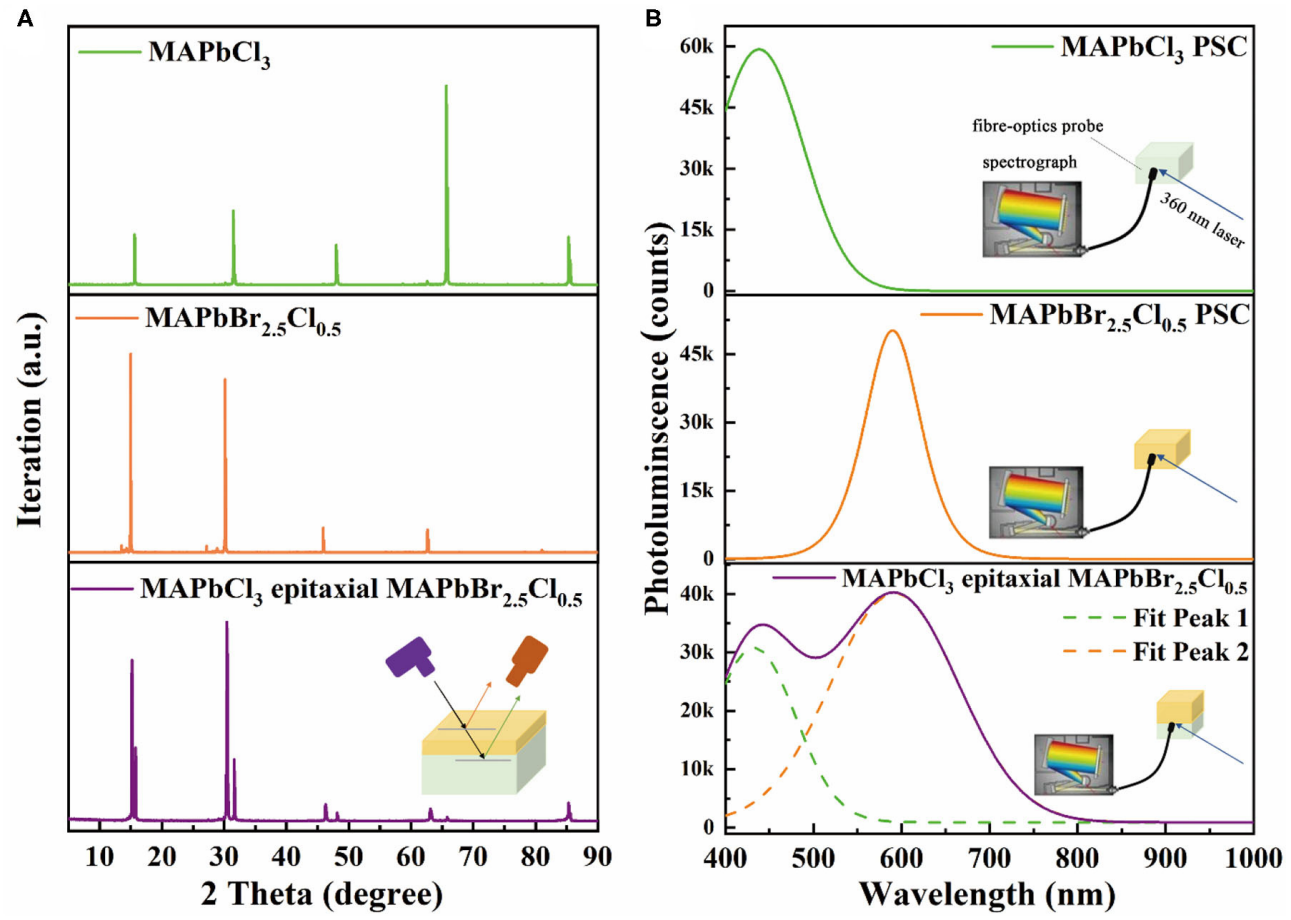
**(A)** XRD spectra and **(B)** PL spectra of MAPbCl_3_ PSC, MAPbBr_2.5_Cl_0.5_ PSC, and MAPbCl_3_ PSC on epitaxial MAPbBr_2.5_Cl_0.5_. Insets: the schematic diagram of PL spectra test.

The comparison between X-ray diffraction (XRD) patterns of MAPbCl_3_ PSC, MAPbBr_2.5_Cl_0.5_ PSC, and MAPbCl_3_ PSC on epitaxial MAPbBr_2.5_Cl_0.5_ are shown in Figure 3A. From the figure, the XRD spectra of the MAPbCl_3_ PSC on epitaxial MAPbBr_2.5_Cl_0.5_ is similar to the MAPbBr_2.5_Cl_0.5_ PSC but shifts slightly to larger angles, due to the combination of MAPbCl_3_ and MAPbBr_2.5_Cl_0.5_. The diffraction peaks in the XRD spectra correspond to the integer of the wavelength, which indicated the one crystalline structure of MAPbCl_3_ PSC on epitaxial MAPbBr_2.5_Cl_0.5_. The series of distinct characteristic peaks and small full width at half-maximum of the MAPbCl_3_ PSC on epitaxial MAPbBr_2.5_Cl_0.5_ indicate the high-quality crystallization. In addition, its diffraction peak positions nearly overlap with MAPbBr_2.5_Cl_0.5_ PSC and MAPbCl_3_ PSC, which corresponds to the reported graded heterojunction (Li et al., 2019b). The existence of main and secondary peaks in the XRD spectra of MAPbCl_3_ PSC on epitaxial MAPbBr_2.5_Cl_0.5_ is attributed to the diffraction of both thin epitaxial MAPbBr_2.5_Cl_0.5_ layer and MAPbCl_3_ layer.

The photoluminescence (PL) spectra of MAPbCl_3_ PSC, MAPbBr_2.5_Cl_0.5_ PSC, and epitaxial MAPbBr_2.5_Cl_0.5_ on MAPbCl_3_ block are displayed in Figure 3B. A 360-nm laser beam is used as the light source and incident on the surface of MAPbCl_3_ PSC, MAPbBr_2.5_Cl_0.5_ PSC, and the interface of the MAPbBr_2.5_Cl_0.5_-MAPbCl_3_ heterojunction. The PL peaks of MAPbCl_3_ PSC on epitaxial MAPbBr_2.5_Cl_0.5_ are similar to the stack of MAPbCl_3_ PSC and MAPbBr_2.5_Cl_0.5_ PSC. From this evidence, it can hereby be concluded that the MAPbBr_2.5_Cl_0.5_ PSC was successfully grown on the MAPbCl_3_ PSC.

To show the effects of reducing lattice mismatch between heterojunction interface by epitaxially combining another kind of PSC and the enhancement of the epitaxial PIN device performance, the spin-coating device with structure Au/poly-TPD/MAPbBr_2.5_Cl_0.5_ PSC/C60/PCBM/Ag was also fabricated for comparison.

**Figure 4:**
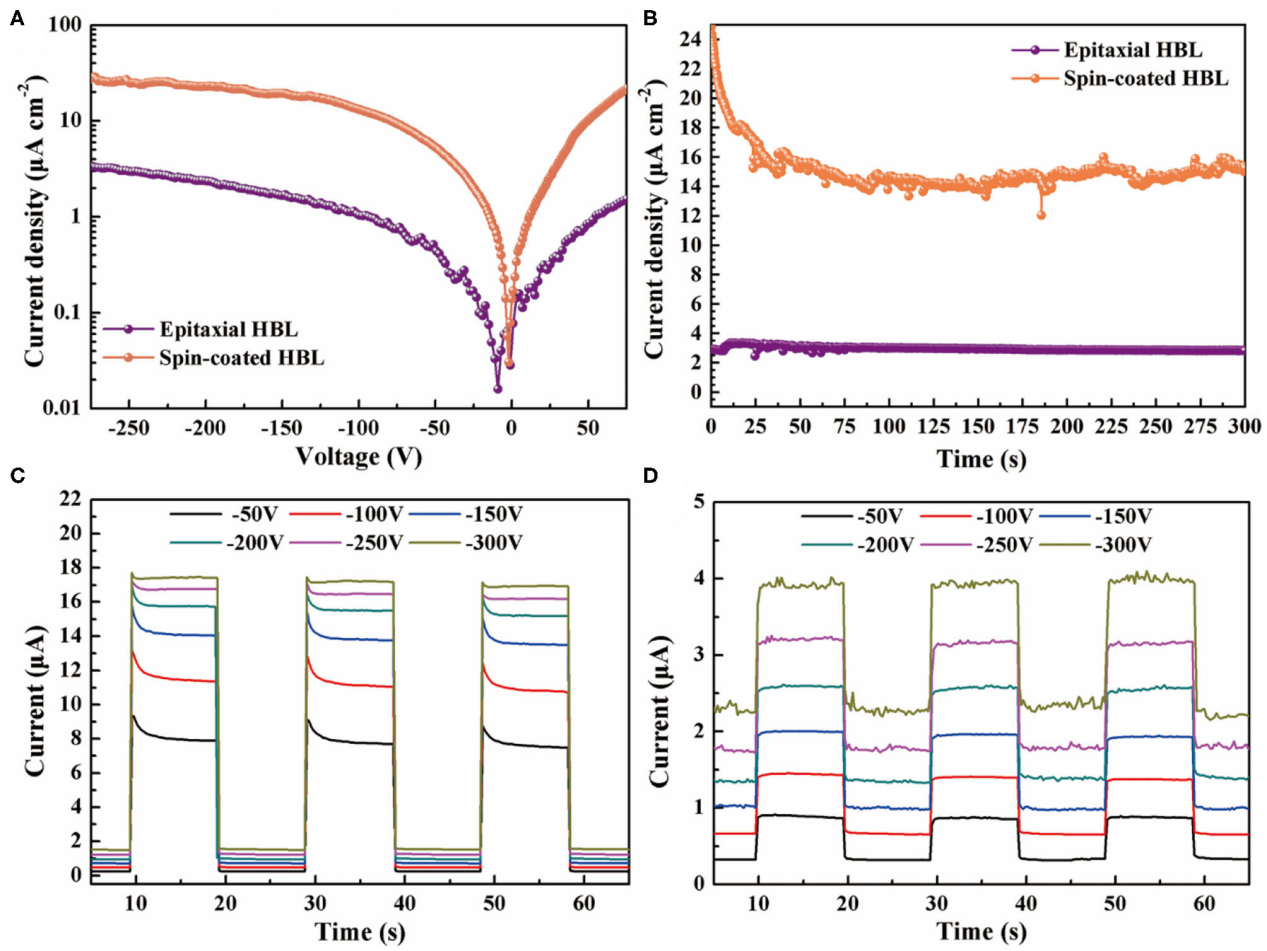
**(A)** Current density as a function of the voltage bias of the epitaxial HBL device and spin-coated HBL device in darkness. **(B)** Long-term dark current stability of the epitaxial HBL device and spin-coated HBL device. Temporal response of the photodiode based on **(C)** the epitaxial HBL device and **(D)** spin-coated HBL device under different voltage bias.

The dark current density–voltage (*J*–*V*) characteristic curves of the epitaxial HBL device and spin-coated HBL device are shown in Figure 4A for comparison. The result indicates that the epitaxial HBL device shows lower dark current density than spin-coated HBL device in reverse voltage. At −250 V, the spin-coated device has a dark current density of 25 μA cm^−2^, which is only 2.9 μA cm^−2^ for the epitaxial HBL device. This indicates that the combination between the intrinsic layer and HBL by epitaxial growth contributes to the reduction of dark current. Furthermore, the dark current density of the epitaxial HBL device shows a value <100 nA cm^−2^ under −20 V, implying that the device has an advantage in the low dark current noise and large dynamic range.

Figure 4B shows the long-term dark current stability of the epitaxial HBL device and spin-coated HBL device under −250 V reverse bias voltage. As shown in the figure, the dark current of the spin-coated HBL device declines quickly in 30 s after startup and takes place at relatively high amplitude fluctuations than the epitaxial HBL device, because the spin-coated organic molecular material is unstable under a high voltage. The amplitude of the vibrated dark current for the epitaxial HBL device is <40 nA cm^−2^ but more than 4 μA cm^−2^ for the spin-coated HBL device after 2 min. The more stable long-term dark current for the epitaxial HBL device can be attributed to the better lattice matched interface between the epitaxial HBL and MAPbBr_2.5_Cl_0.5_ PSC.

The photon detection performance is measured by a temporal photocurrent at −50 V, −100 V, −150 V, −200 V, −250 V, and −300 V, on 460-nm and 130-μW blue light illumination, as shown in Figures 4C,D. All the photo responses of the epitaxial HBL device are greater than the spin-coated HBL device under different bias voltages. The rise of the photo response rate is higher than dark currents for the epitaxial HBL device with increasing bias voltage. The responsivities of the epitaxial HBL device are calculated as:

(1) $$R=\left(I_{photo}-I_{dark}\right)/P,$$

where *P* is incident optical power, which is 58.7 mA W^−1^, 81.7 mA W^−1^, 98.6 mA W^−1^, 112 mA W^−1^, 117 mA W^−1^, and 122 mA W^−1^ from −50 to −300 V, respectively. The larger responsivity is caused by the decrease of carrier recombination on the MAPbCl_3_/MAPbBr_2.5_Cl_0.5_ heterojunction interface.

**Figure 5:**
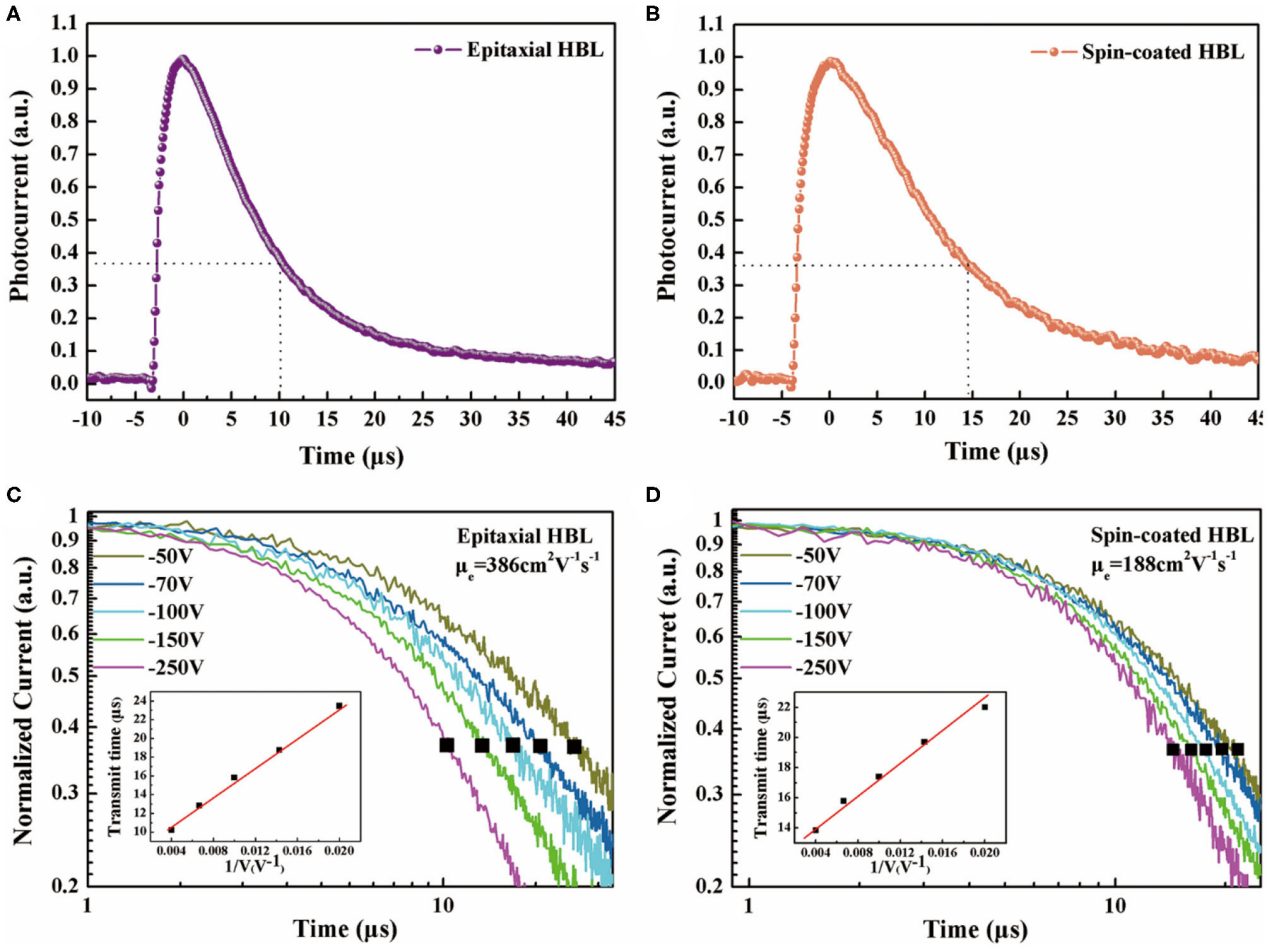
Normalized transient current response of the **(A)** epitaxial EBL device and **(B)** spin-coated EBL device. Decay curve of the **(C)** epitaxial EBL device and **(D)** spin-coated EBL device. Insets: electron transmit time vs. the reciprocal of bias (the solid line is a linear fit to the data).

Response speed is a significant factor for photodiodes that are applied in detection and imaging. In this study, a 365-nm pulsed laser with 7 ns pulse width at 20 Hz frequency is used as an excitation light source to measure the decay process of the photocurrent that reflects the detection speed of the device. Figures 5A,B show the decay process of the photodiodes with epitaxial HBL and spin-coated HBL, having 5.3 and 3.2 mm thickness, respectively, under −250 V bias. The fall time defined as decaying to e^−1^ of the maximum for different devices are measured as 10 and 15 μs, respectively, which signify that the photodiode with epitaxial HBL has a faster detection speed. Subsequently, the carrier mobilities of the epitaxial HBL and spin-coated devices are measured using the time of flight (TOF) technique. The average charge-carrier mobilities of the epitaxial HBL and spin-coated HBL devices are measured by the 365-nm nanosecond laser illuminated from the Au electrode side. The transient time for electron carrier transport through the whole perovskite device was used for average electron mobility calculation according to the formulation (Wei et al., 2016; Thirimanne et al., 2018; Hu et al., 2020):

(2) $$\begin{array}{l} \mu \;=\;d^{2}/V\tau \text{,} \end{array}$$

where μ is the mobility, *d* is the thickness of the device, τ is the transmit time, and *V* is the bias voltage. The transient photocurrent responses of the epitaxial device and spin-coated device under different bias voltages were recorded and are shown in Figures 5C,D. By fitting the plot of τ-V^−1^, as shown in the insets, the calculated results reveal that the average electron mobility of the spin-coated HBL device is 188 cm^2^ V^−1^ s^−1^ while that of the epitaxial HBL device is 386 cm^2^ V^−1^ s^−1^. The epitaxial HBL device has better average electron mobility than the spin-coated HBL device, which is attributed to the substitution low surface trap single crystals for additional solution-processed layers. A detailed comparison of our epitaxial HBL perovskite photodetector with the reported layered perovskite-based photodetectors is summarized in Table 1.

**Table 1:** Comparison of the epitaxial HBL perovskite photodetector with the reported layered perovskite photodetectors.

**Device structure**	**Responsivity (mA W^−1^) @ applied electric field, light source**	**Decay time (μs) @ applied electric field**	**References**
Au/poly-TPD/MAPbBr_3_ PSC/C60 doped PCBM/Ag	1.252 @ 0.003 V/μm,532 nm light	26 @ 0.015 V/μm	Wang et al., 2018
Glass/SnO_2_/PCBM/MAPbI_3_/Au	11.8 @ 0.05 V/μm,460 nm light	63.3 @ 0.05 V/μm	Su et al., 2019
FTO/TiO_2_/MAPbI_3_/ PDCBT or Spiro-OMeTAD/Au	92 @ 0 V/μm, 405 nm light 132 @ 0.3 V/μm, 620 nm light	210 @ 0 V/μm 140 @ 0.3 V/μm	Tang et al., 2019
ITO/MAPbI_3_/PCBM/C60/Ag	35 @ 0.066 V/μm, white light	227 @ 0.066 V/μm	Lv et al., 2018
Au/poly-TPD/MAPbBr_2.5_Cl_0.5_-MAPbCl_3_/Ag	117 @ 0.047 V/μm, 460 nm light	10 @ 0.047 V/μm	This study

**Figure 6:**
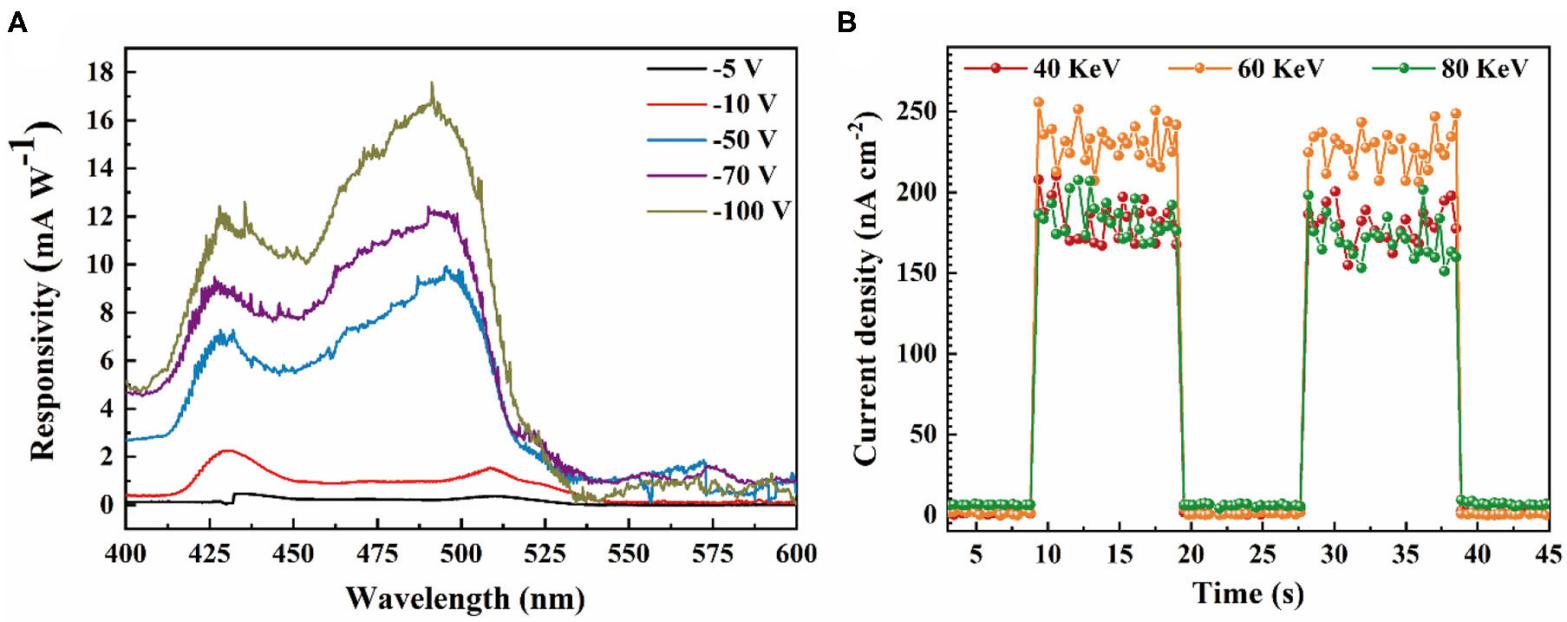
**(A)** Responsivity spectra of the epitaxial PIN device under different voltage bias. **(B)** Temporal response of the epitaxial PIN device at 0 V bias with different energy X-rays on and off.

Furthermore, other key parameters of our epitaxial photodiode were also measured. When light beam with wavelength that varies continuously is incident on the cathode of the photodiode with epitaxial HBL, its responsivity spectra under different voltages are shown in Figure 6A. The results show a few smaller responsivity than that of temporal response in Figure 4C because incident light was partially obscured by the thick Ag electrode. The photodiode is most sensitive at 430 and 500 nm but almost insensitive for photons with wavelength exceeding 540 nm. With the increase of reverse voltage, the responsivity improves significantly. This is due to the different band gap for MAPbCl_3_ PSC and MAPbBr_2.5_Cl_0.5_ PSC, i.e., 3.1 and 2.26 eV, respectively. In addition, short-wave photons generate a higher current response (2.26 mA W^−1^ at 430 nm, 1.54 mA W^−1^ at 510 nm) under low-bias voltage (−10 V). However, long-wave photons generate a higher current response (12.24 mA W^−1^ at 430 nm, 16.73 mA W^−1^ at 500 nm) under high-bias voltage (−100 V). This is because electron–hole pairs are generated at a deeper depth inside the active layer with the increase of the incident photon wavelength. Electron–hole pairs at different depths have been collected by applying different reverse voltages. The collection efficiency has also been improved with increase of the applying voltage. The noise current (*I*_n_) of the epitaxial device under −100 V is measured as 1.12 × 10^−10^ A Hz^−1/2^ approximately. Based on the *I*_n_ and *R*, the specific detectivity (D^*^) of the epitaxial device could be obtained by the following equations (Li et al., 2020):

(3) $$D^{*}=R\times \sqrt{Af}/I_{n},$$

where *A* is the active layer area and *f* is the bandwidth. The results of D^*^ value is similar to the trend of *R* (Li et al., 2019a). The maximum D^*^ of the epitaxial device is calculated about 7.32 × 10^7^ cm Hz^1/2^ W^−1^ at 500 nm under −100 V. The external quantum efficiency (EQE) of the epitaxial device under −100 V at 500 nm is calculated as about 4.5% by the following equations:

(4) EQE=R×hc/qλ,

where *h* is Plank's constant, *c* is the velocity of light, *q* is the absolute value of the electron charge, and λ is the light wavelength.

Due to the thickness of the epitaxial photodiode in the solution process being suitable for the potential application of X-ray detection, the X-ray detection performance under zero bias is also investigated in this work. Photocurrents caused by 40-keV, 60-keV, and 80-keV X-ray photons with a dose rate 27.91 μGy_air_ s^−1^, 62.03 μGy_air_ s^−1^, and 84.89 μGy_air_ s^−1^, respectively, are shown in Figure 6B. The 5.32-mm-thick device with epitaxial EBL with self-powered shows a satisfactory response to the 40-keV, 60-keV, and 80-keV X-ray photons due to the built-in electric field of the PIN device. The X-ray detection sensitivities (*S*) of the epitaxial HBL device are calculated as:

(5) $$\begin{array}{l} S=Q/AX\text{,} \end{array}$$

where *Q* is the collected photo charge, *A* is the effective detection area, and *X* is the radiation dose, which is 7.08 × 10^3^ μC Gy_air_^−1^ cm^−2^, 4.04 × 10^3^ μC Gy_air_^−1^ cm^−2^, and 2.38 × 10^3^ μC Gy_air_^−1^ cm^−2^ for the 40-, 60-, and 80-keV X-ray photons, respectively. The sensitivity of our epitaxial device with self-powered is superior to the majority of hybrid perovskite film and single crystalline device, such as MAPbI_3_ film device (25 μC Gy_air_^−1^ cm^−2^) (Yakunin et al., 2015), CsPbBr_3_ thick film device (0.27–1.7 × 10^3^ μC Gy_air_^−1^ cm^−2^) (Gou et al., 2019), and MAPbBr_3_ single crystalline device (80 μC Gy_air_^−1^ cm^−2^) (Wei et al., 2016). Moreover, the sensitivity of our epitaxial device with self-powered is stronger than that of the currently commercial detectors such as a-Se, CdZnTe, HgI_2_, and PbI_2_ working at a much higher field (Schieber et al., 2001). The results reveal the advantage of the application of our epitaxial PIN photodiode for X-ray detection.

## Conclusion

To summarize, heterojunction bulk PSC has been fabricated based on liquid-phase epitaxial MAPbBr_2.5_Cl_0.5_ on MAPbCl_3_ PSC. A 10-fold reduction of the dark current is achieved for the PIN photodiode with epitaxial HBL in comparison to the photodiode fabricated by spin-coated additional organic n-type semiconductor materials as HBL. Additionally, the epitaxial HBL device also shows greater responsivity, better stability, faster response time to 10 μs, and a higher average charge-carrier mobility up to 386 cm^2^ V^−1^ s^−1^ than the spin-coated HBL device. The device also reveals a satisfactory detection sensitivity up to several mC Gy_air_^−1^ cm^−2^ for 40- to 80-keV X-ray photons. This research proves that epitaxial growth is a more reliable, effective method to fabricate perovskite–perovskite heterojunctions and can avoid lattice mismatch on the interface. Hence, the use of epitaxial PSC HBL to form heterojunction interface can be a promising method for the fabrication of high-performance perovskite photodiode devices.

## Data Availability Statement

The raw data supporting the conclusions of this article will be made available by the authors, without undue reservation.

## Author Contributions

YP, XW, and YX grew the perovskite single crystals. YP conducted the epitaxial experiments and wrote this manuscript. YP and YL conducted the measurements. XW and YP analyzed the results. All authors have made comments on the manuscript.

## Conflict of Interest

The authors declare that the research was conducted in the absence of any commercial or financial relationships that could be construed as a potential conflict of interest.
